# Tenant Right-to-Counsel and Adverse Birth Outcomes in New York, New York

**DOI:** 10.1001/jamapediatrics.2024.4699

**Published:** 2024-10-28

**Authors:** Kathryn M. Leifheit, Katherine L. Chen, Nathaniel W. Anderson, Cecile Yama, Achyuth Sriram, Craig Evan Pollack, Alison Gemmill, Frederick J. Zimmerman

**Affiliations:** 1 Department of Pediatrics, David Geffen School of Medicine, University of California, Los Angeles; 2 Division of General Internal Medicine & Health Services Research, David Geffen School of Medicine, University of California, Los Angeles; 3 Department of Health Policy & Management, Fielding School of Public Health, University of California, Los Angeles; 4 National Clinician Scholars Program, David Geffen School of Medicine, University of California, Los Angeles,; 5 Department of Health Policy & Management, Bloomberg School of Public Health, Johns Hopkins University, Baltimore, Maryland; 6 School of Nursing, Johns Hopkins University, Baltimore, Maryland; 7 Department of Population, Family and Reproductive Health, Bloomberg School of Public Health, Johns Hopkins University, Baltimore, Maryland; 8 Department of Urban Planning, Luskin School of Public Affairs, University of California, Los Angeles

## Abstract

**Importance:**

In 2017, New York, New York, launched the United States’ first right-to-counsel program, guaranteeing lawyers to low-income tenants in select zip codes, which was associated with reducing eviction risk by half. Given documented associations between evictions during pregnancy and adverse birth outcomes, the right-to-counsel program may be associated with improved birth outcomes.

**Objective:**

To measure associations between zip code–level right-to-counsel access and risk of adverse birth outcomes, including preterm birth and low birth weight, among infants born to Medicaid-insured birthing parents.

**Design, Setting, and Participants:**

This retrospective cohort study leveraged the staggered rollout of New York’s right-to-counsel program from January 2016 to February 2020 as a natural experiment using a population-based sample of live births to Medicaid-insured birthing parents residing in New York, New York. Data were analyzed from February 2022 to September 2024.

**Exposure:**

Zip code right-to-counsel status 9 months prior to birth.

**Main Outcomes and Measures:**

Adverse birth outcomes were measured using individual birth records from the New York Bureau of Vital Statistics. Outcomes included dichotomous indicators of low birth weight (<2500 g), preterm birth (<37 weeks’ gestation), and a composite of both. Difference-in-differences linear probability models controlled for year, month, and zip code and included clustered standard errors.

**Results:**

Among 260 493 live births (mean [SD] birthing parent age, 29 [6] years) from January 2016 to February 2020, 43 081 births (17%) were to birthing parents residing in zip codes where right-to-counsel was available during pregnancy. Exposure to right-to-counsel during pregnancy was associated with statistically significant reductions in infants’ probability of adverse birth outcomes, with reductions of 0.73 (95% CI, 0.06-1.41) percentage points in low birth weight, 0.91 (95% CI, 0.10-1.71) percentage points in preterm birth, and 0.96 (95% CI, 0.09-1.84) percentage points in the composite outcome in treated vs untreated zip codes.

**Conclusions and Relevance:**

This cohort study found that right-to-counsel was associated with reduced risk of adverse birth outcomes among Medicaid-insured birthing parents. These findings suggest that eviction prevention via right-to-counsel may have benefits that extend beyond the courtroom and across the life-course.

## Key Points

**Question:**

Was eviction prevention via free legal representation in housing court (right-to-counsel) in New York, New York, associated with birth outcomes in Medicaid-insured birthing parents?

**Findings:**

In this cohort study including 260 493 live births, right-to-counsel was associated with a statistically significant 0.96–percentage point absolute reduction in adverse birth outcomes among Medicaid-insured birthing parents.

**Meaning:**

These findings suggest that right-to-counsel programs may lead to improvements in key population health indicators.

## Teaser

This cohort study examines the associations of zip code–level right-to-counsel access with risk of adverse birth outcomes, including preterm birth and low birth weight, among infants born to Medicaid-insured birthing parents in New York, New York.

## Introduction

Evictions are common among low-income renters and contribute to poor health. Renters with children are at particular risk, with 10.4% receiving an eviction filing each year, compared with a rate of 5.0% among those without children.^1^ A large and evolving literature documents adverse health effects of evictions.^1,2,3,4,5^ Evictions impose significant stress,^6,7^ disrupted health care access,^8^ and nutritional deficits.^9^ Pregnancy is a sensitive period during which adversity can pose especially strong risk to pregnant people’s health and to fetal development.^10^ Consequently, evictions among pregnant people are associated with adverse birth outcomes.^11^ A study linking eviction data to birth certificates in the state of Georgia found that evictions during pregnancy were associated with increases in infants’ probability of low birth weight and, separately, preterm birth.^12^ By extension, interventions to prevent evictions might improve birth outcomes.

Tenant right-to-counsel is one such preventive measure. Right-to-counsel programs provide legal representation to tenants facing evictions. In contrast to criminal cases, defendants in civil cases, such as eviction hearings, are not guaranteed a lawyer under federal law. As a result, there is a power imbalance in rent courts: in 2024 data collected from 39 jurisdictions (states and large cities) spread across the country, 83% of landlords had legal representation in eviction cases, while only 4% of tenants did.^13^ Lacking lawyers, tenants are less able to assert their due process, win cases, or reach fair settlements with landlords. Additionally, many tenants do not appear in courts and are evicted via default judgements.

New York, New York, launched the United States’ first right-to-counsel program in 2017, providing attorneys to tenants earning below 200% of the federal poverty level through an initiative known as Universal Access to Legal Services.^14^ New York’s program was rolled out in phases by zip code, prioritizing neighborhoods with high eviction rates and low tenant representation. The program began with 10 zip codes in winter 2017, adding new zip codes over time. In March 2020, in response to sudden increases in economic and housing strain during the COVID-19 pandemic, the program was expanded ahead of schedule to cover low-income renters across the entire city.^14^ Tenants were screened for eligibility by program staff and assigned a lawyer on the day of their first appearances in court.^14^ Previous evaluations suggest that the program has doubled rates of legal representation in eviction cases.^15^ Right-to-counsel access has also dramatically reduced evictions: in one study, living in a right-to-counsel zip code was associated with a 45% to 78% reduction in the likelihood that an eviction case would result in an eviction warrant (ie, a judge’s orders triggering forcible removal of a tenant).^16^ Qualitative research finds that right-to-counsel may improve health by helping tenants maintain housing, mitigating harms associated with evictions (eg, securing extra time for a move, reducing rent owed, or lowering stress during an eviction), and empowering renters to organize.^17^ In this analysis, we aimed to determine whether New York’s right-to-counsel program was associated with reduced incidence of adverse birth outcomes (preterm birth and adverse birth outcomes) among low-income residents of New York, New York.

## Methods

This cohort study was reviewed and approved by the University of California, Los Angeles, institutional review board. The University of California, Los Angeles, institutional review board waived the requirement for informed consent, assent, and parental permission under 45 CFR 46.116, as it was a secondary data analysis posing minimal risk of harm to participants. This study is reported following the Strengthening the Reporting of Observational Studies in Epidemiology (STROBE) reporting guideline.

We performed a cohort study leveraging New York’s staggered implementation of right-to-counsel from 2017 to 2020 as a natural experiment. During this study period, 9 evictions were filed per 100 renter households in New York.^18^ Based on national trends in evictions by income and family structure,^1^ it is reasonable to assume that this filing rate was 2 to 3 times higher among low-income birthing people in New York.

### Data and Study Population

We obtained birth certificate data on all live births occurring in New York from 2016 to 2020 from the New York City Department of Health and Mental Hygiene’s Bureau of Vital Statistics. Each record contains information pertaining to birth month, parental and infant demographics and health status, and zip code of residence at the time of birth. For the present study, we limited our sample to infants born prior to March 2020 to exclude time that was covered by the COVID-19 pandemic, when the State of New York and federal government enacted temporary eviction moratoria. To match right-to-counsel inclusion criteria, we further limited our sample to infants whose parents resided within New York, New York, and who had Medicaid insurance (a proxy for low income; Medicaid income limits for pregnant people and infants were set at 223% of the federal poverty level during the study period^19^). Finally, we restricted the sample to infants with complete data on gestational age and birth weight.

### Definition of Outcomes

Our primary outcome was adverse birth outcomes, a composite, dichotomous indicator of whether the child was born either with less than 2500 g birth weight or at less than 37 weeks’ gestation. We also examine each component indicator (low birth weight, preterm birth) as unique outcomes.

### Specification of Right-to-Counsel Treatment

**Figure 1.**
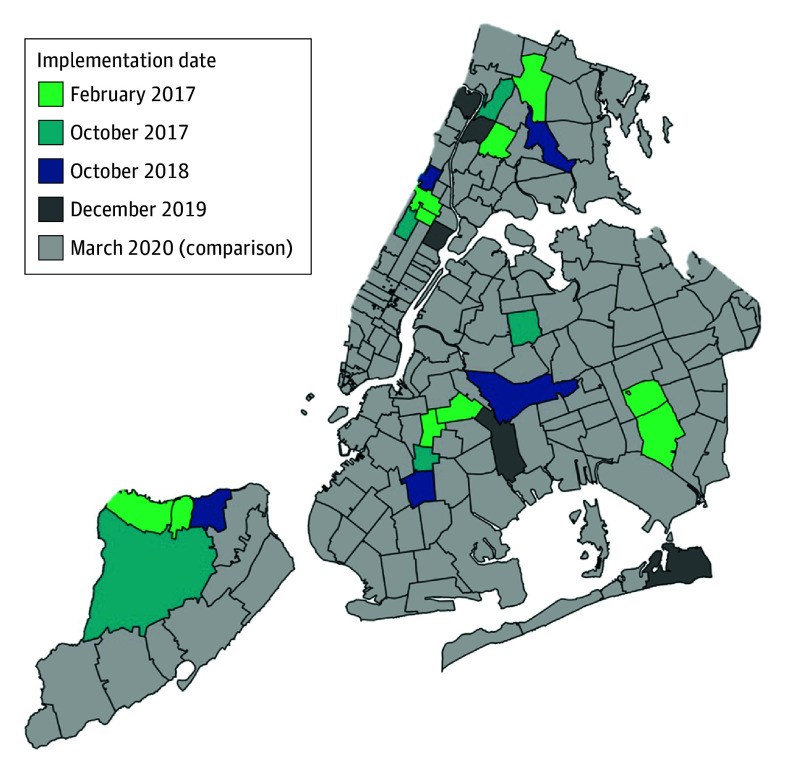
Right-to-Counsel Implementation Timing by New York, New York, Zip Code

We specified zip code–specific dates of right-to-counsel implementation based on reports from New York’s Office of Civil Justice. Dates were further validated via conversations with the Office of Civil Justice, people providing legal services via right-to-counsel (ie, lawyers and legal teams) in New York, and tenant advocates. Implementation was defined as the date on which essential programmatic elements (eg, courthouse-based tenant outreach) were in place and accessible to tenants. Dates used were broadly consistent with previous studies.^15,16^ Figure 1 maps the staggered rollout of right-to-counsel in New York.

We lagged the policy treatment by 9 months to ensure that an infant’s full gestational period was covered by right-to-counsel. For example, an infant residing in a zip code where right-to-counsel was implemented in October 2017 would be counted as treated if they were born during or after July 2018.

### Statistical Analysis

We first compared sociodemographic characteristics of birthing parents in zip codes ever vs never treated with right-to-counsel. We then plotted incidence of adverse birth outcomes over time in 2 groups: infants in zip codes that never received the intervention during the study period (never treated) and infants in zip codes that eventually received the intervention (ever-treated).

Second, we compared raw incidence of outcomes before and after policy implementation in zip codes with right-to-counsel, compared with never-treated zip codes. Given the staggered rollout of right-to-counsel, we conducted these comparisons separately for each implementation phase.

We then used difference-in-difference models to estimate the difference between the pre-post change in adverse birth outcomes observed in zip codes treated with right-to-counsel vs the change expected had those zip codes never been treated.^20^ We estimated this counterfactual using data from zip codes that did not receive right-to-counsel during the study period. The validity of this approach relies on a parallel-trends assumption, ie, that treated and untreated groups would have parallel or common trends in the absence of treatment. We tested this assumption using an event-study specification,^21^ where the strength of associations is allowed to vary over time, relative to treatment. Additional details on our model specification, including assumption checking, can be found in the eAppendix in Supplement 1.

We used linear probability difference-in-differences models to measure associations between right-to-counsel and adverse birth outcomes.^22^ Treatment was specified as a time-varying, lagged indicator of whether an infant’s zip code was treated with right-to-counsel 9 months prior to birth. The indicator is coded zero for never-treated zip codes and pretreatment zip codes; it is coded 1 after treatment. To control for seasonal variation in birth outcomes and time-invariant differences between treated and untreated zip codes,^23^ we adjusted for year, month, and zip code. We used a robust variance estimator to account for correlation due to clustering of individuals within zip codes.

We conducted a number of sensitivity analyses. To address the possibility that zip code–level demographic changes concurrent with right-to-counsel rollout might confound our results, we ran a difference-in-differences model adjusted for the following individual-level covariates: birthing parent age, race and ethnicity, education, nativity, marital status, and parity (ie, a birthing parent’s cumulative number of live births). Additionally, we tested 2 alternate exposure definitions: percentage of gestation exposed to right-to-counsel based on estimated date of conception and first trimester of right-to-counsel exposure. These 2 alternate exposure models included gestations that were partially exposed to right-to-counsel in the treatment group, whereas our main models required treatment for the full gestational period. We also ran models with birth weight and gestational age specified continuously, as well as models using more extreme outcomes (very low birth weight, defined as <1500 g, and very preterm, defined as <32 weeks). Finally, to explore potential heterogeneity by treatment phase, we estimated models separately by phase and ran heterogeneity-robust models following the Callaway and Sant’Anna method for differences in differences with multiple periods.^24^ Because there are large racial and ethnic disparities in eviction risk,^1^ equitable rollout of right-to-counsel might especially benefit the health of populations that are disproportionately evicted (in particular, infants born to non-Hispanic Black birthing parents). To explore potentially heterogeneous policy impacts between racial and ethnic groups, we stratified models by birthing parent race and ethnicity, focusing on Hispanic parents and non-Hispanic Asian or Pacific Islander, Black, and White groups for adequate sample sizes. Our analyses were not adequately powered to test for effect modification via statistical interaction.

All statistical analyses were conducted in Stata software version 17.0 (StataCorp). *P* values were 2-sided, and statistical significance was set at *P* ≤ .05. Data were analyzed from February 2022 to September 2024.

## Results

### Study Population and Descriptive Results

There were 479 617 live births in New York between January 2016 and February 2020 (eFigure 1 in Supplement 1). Among these, 48 241 birthing parents (10%) resided outside New York and were excluded. An additional 170 870 births (40% of New York resident births) were not Medicaid insured and were excluded. Finally, 13 births (<1% of Medicaid-insured births) were missing gestational age or birth weight and were excluded. Ultimately, our study sample included 260 493 infants (mean [SD] birthing parent age, 29 [6] years), among whom 43 081 births (17%) were to birthing parents living in zip codes ever treated with right-to-counsel. Birthing parents in zip codes ever vs never treated by right-to-counsel during the study period had similar age distributions but were more likely to be Hispanic or non-Hispanic Black, less likely to be US born or married, and more likely to be having their first child (Table 1).

**Table 1.** Birthing Parent and Infant Characteristics by Zip Code Right-to-Counsel Status

Birthing parent characteristics	Births by zip code–level right-to-counsel, No. (%)		*P* value
	Never (n = 217 412)	Ever (n = 43 081)	
Age, mean (SD), y	29 (6)	29 (6)	.11
Race and ethnicity			
Hispanic	78 671 (36)	20 246 (47)	<.001
Non-Hispanic Asian and Pacific Islander	39 952 (18)	5254 (12)	
Non-Hispanic Black	45 406 (21)	12 393 (29)	
Non-Hispanic White	50 634 (23)	4630 (11)	
Another race or ethnicity^a^	2749 (1)	558 (1)	
<High school education	55 892 (26)	11 618 (27)	<.001
US-born	86 371 (4)	16 366 (38)	<.001
Married	111 588 (51)	17 763 (41)	<.001
Nulliparous	78 183 (36)	16 189 (38)	<.001

^a^ Includes people who identify as American Indian and Alaska Native, people with 2 or more races, and people who did not list a race or ethnicity

**Figure 2.**
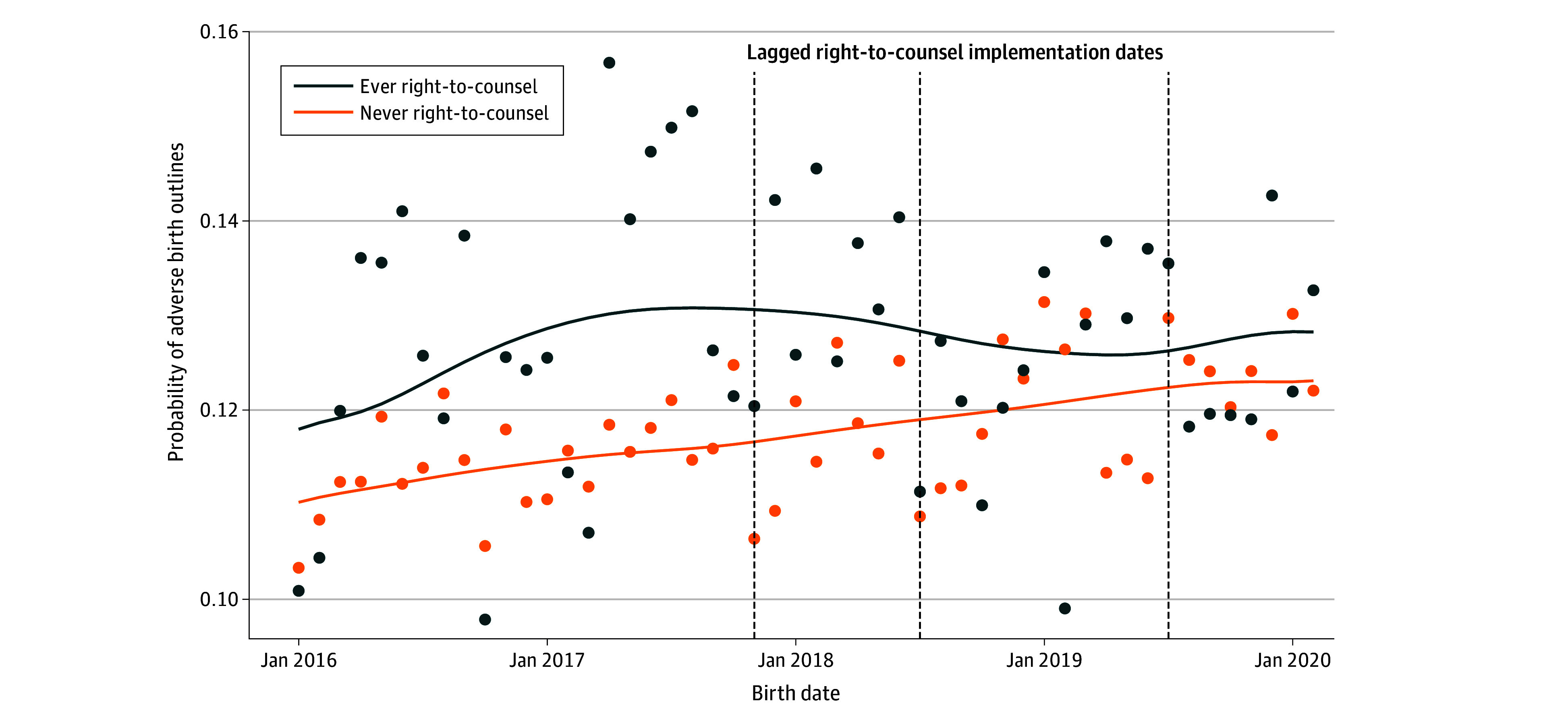
Trends in Adverse Birth Outcomes Within Right-to-Counsel Ever vs Never Treatment Groups Points indicate monthly incidence of adverse birth outcomes across groups of zip codes treated and untreated by right-to counsel. Lines indicate nonparametric smoothed trends. Implementation dates are lagged by 9 months to show the first month at which a birth is considered treated by the policy

A descriptive plot of birth outcomes over time (Figure 2) in our sample of Medicaid births showed that the incidence of adverse birth outcomes was higher at baseline in right-to-counsel zip codes compared with those never exposed to right-to-counsel, and the incidence increased steadily in both groups. Following right-to-counsel implementation, adverse birth outcomes leveled off in treated zip codes, while they continued to increase in never-treated zip codes.

### Difference-in-Differences Results

We first present unadjusted frequencies of adverse birth outcomes by right-to-counsel treatment group and time (pretreatment vs posttreatment) in Table 2. Across all treatment groups and outcomes, we saw that incidence of outcomes either remained stable or decreased in treated zip codes, while increasing over time in zip codes never treated with right-to-counsel. As a result, unadjusted difference-in-difference coefficients were consistently negative, suggesting a protective association.

**Table 2.** Unadjusted Frequences of Adverse Birth Outcomes by Zip Code Right-to-Counsel Treatment Group and Time, Including Difference-in-Difference Results

Treatment group	Births, No. (%)		Difference, percent points	Difference-in-differences, percentage points
	Pre–right-to-counsel	Post–right-to-counsel		
**Composite outcome**				
February 2017				
Treated	1246 (13.72)	1366 (13.44)	−0.28	−0.82
Never treated	11 532 (11.45)	13 992 (11.99)	0.54	
October 2017				
Treated	888 (11.65)	512 (11.43)	−0.22	−0.81
Never treated	15 580 (11.52)	9944 (12.11)	0.59	
October 2018				
Treated	1261 (12.60)	209 (12.13)	−0.47	−1.27
Never treated	21 458 (11.62)	4039 (12.42)	0.80	
**Low birth weight**				
February 2017				
Treated	899 (9.90)	1005 (9.89)	−0.01	−0.39
Never treated	8252 (8.19)	10 000 (8.57)	0.38	
October 2017				
Treated	631 (8.28)	351 (7.83)	−0.45	−0.84
Never treated	11 160 (8.25)	7092 (8.64)	0.39	
October 2018				
Treated	890 (8.89)	141 (8.18)	−0.71	−1.18
Never treated	15 391 (8.32)	2861 (8.79)	0.47	
**Preterm birth**				
February 2017				
Treated	939 (10.34)	1033 (10.16)	−0.18	−0.73
Never treated	8670 (8.61)	10 689 (9.16)	0.55	
October 2017				
Treated	697 (9.14)	397 (8.86)	−0.28	−0.80
Never treated	11 780 (8.71)	7579 (9.23)	0.52	
October 2018				
Treated	1011 (10.10)	159 (9.23)	−0.87	−1.55
Never treated	16 276 (8.80)	3083 (9.48)	0.68	

**Figure 3.**
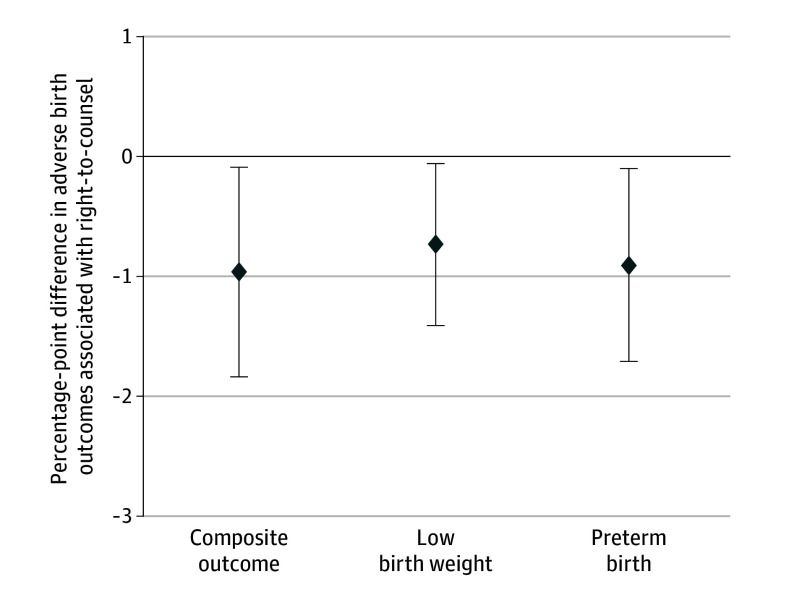
Difference-in-Differences Results Comparing Change in Probability of Adverse Birth Outcomes Between Medicaid-Insured New York City Infants in Zip Codes With vs Without Right-to-Counsel, January 2016 to February 2020 (N = 260 493) Results are based on difference-in-difference linear probability models that control for month, year, and zip code, with SEs clustered by zip code. Coefficients represent differences in adjusted pre-post right-to-counsel differences in incidence of adverse birth outcomes, comparing births in treated vs untreated zip codes. Findings are statistically significant (composite outcome: *P* = .03; low birth weight: *P* = .03; preterm birth: *P* = .03).

Visual inspection of the event study plot (eFigure 2 in Supplement 1) did not indicate any violations of the parallel-trends assumption, allowing us to proceed with difference-in-differences models. Difference-in-differences results were consistent with unadjusted results. Coefficients presented in Figure 3 should be interpreted as adjusted, weighted means of the difference-in-difference estimates presented in Table 2. Incidence of the composite adverse birth outcome was 0.96 (95% CI, 0.09 to 1.84) percentage points lower in zip codes with vs without right-to-counsel (Figure 3). When analyzed separately, associations with low birth weight and preterm birth were also statistically significant, with a reduction of 0.73 (95% CI, 0.06 to 1.41) percentage points in low birth weight and a reduction of 0.91 (95% CI, 0.10 to 1.71) percentage points in preterm birth.

### Sensitivity Analyses

Results from our various sensitivity analyses were consistent with results from our primary analysis (eTable in Supplement 1). Covariate adjustment produced very similar, still statistically significant estimates. Alternate exposure definitions lend suggestive evidence that earlier treatment with right-to-counsel (ie, exposure beginning before or during the first trimester) was more strongly associated with improved outcomes (eTable in Supplement 1). Models assessing birth weight and gestational age as continuous variables did not find statistically significant changes associated with right-to-counsel. Models of very low birth weight and very preterm birth produced null results. Although treatment phase–specific analyses found qualitatively similar associations across phases, heterogeneity-robust results suggest that there may be meaningful heterogeneity in associations between phases.

In models stratified by race and ethnicity, we found right-to-counsel implementation was associated with a reduction in adverse birth outcomes of 1.24 (95% CI, 0.04 to 2.44) percentage points among Hispanic birthing parents, with no statistically significant changes measured among non-Hispanic Asian and Pacific Islander, non-Hispanic Black, and non-Hispanic White birthing parents (eTable in Supplement 1).

## Discussion

This cohort study found that the implementation of right-to-counsel for low-income tenants in New York, New York, was associated with a 0.96–percentage point reduction in adverse birth outcomes among infants born to Medicaid-insured parents. At the population level, reducing the rate of adverse birth outcomes by 0.96 percentage points in a city with more than 60 000 Medicaid-insured births per year amounts to 600 fewer infants born either preterm or with low birth weight each year in New York. Given the persistent ways that birth outcomes impact long-term health and well-being,^25,26,27^ these health benefits will be compounded across the life-course. Additionally, with each preterm birth incurring more than $80 000 in societal costs,^28^ each year of right-to-counsel could translate to nearly $50 million in savings with each annual birth cohort. Our findings build on an evolving literature linking social policies, such as income supports, to improved birth outcomes.^29,30,31^

This finding is particularly noteworthy given that our study estimates health impacts on the entire community of low-income birthing parents, not just those facing eviction. It is likely that our study underestimates the health benefits of right-to-counsel among the subset of birthing parents who were directly affected by the threat of eviction and received counsel. At the same time, the impact of right-to-counsel may not be limited to those immediately threated by eviction: prior research suggests that health benefits of eviction prevention might extend to the broader community through a variety of social, behavioral, and biological pathways.^32^

Sensitivity analyses reinforced our main findings. Null results in models of very low birth weight and very preterm birth suggest that right-to-counsel might not equally impact the full distribution of birth weight and gestational age outcomes. Rather, impacts are likely concentrated near the thresholds for low birth weight and preterm birth.

Since New York’s program launched in 2017, the national movement for right-to-counsel has gained momentum. An additional 17 cities or counties and 5 states have enacted right-to-counsel laws,^33^ and several more are actively considering bills. Prior research has found that cost savings associated with homelessness diversion vastly exceed costs of legal representation.^34^ Our findings suggest that a reduction in adverse birth outcomes should be incorporated into these types of cost-benefit analyses.

### Limitations

Our study has several limitations. First, we assigned right-to-counsel exposure according to zip code at birth. If a parent moved zip codes between conception and birth, right-to-counsel exposure might be misclassified. Given limited data on within-city mobility patterns, it is difficult to gauge the extent or direction of bias this misclassification might introduce into results. Second, as with all quasi-experimental studies, there is a possibility of confounding by concurrent interventions. However, we queried New York policy advocates and are not aware of any zip code–level, health-relevant interventions implemented concurrently with right-to-counsel. Third, we are aware that New York’s Office of Civil Justice offered some level of legal representation in non–right-to-counsel zip codes prior to official right-to-counsel implementation. The existence of right-to-counsel services in control zip codes could bias our results toward the null. Fourth, results may not be generalizable to jurisdictions other than New York implementing right-to-counsel. Fifth, sensitivity analyses suggest that there may have been heterogeneity in treatment effects between implementation phases. Future work should explore heterogeneity in effects as the New York program evolves and new versions arise in other locations. Sixth, we restricted our analyses to live births; this may result in selection bias if right-to-counsel impacts infants’ likelihood of survival.^35^ Future analyses should measure the associations of right-to-counsel policy with infant mortality. Additionally, despite evidence that the impact of right-to-counsel programs on evictions (and, by extension, health) might vary among communities with different racial and ethnic composition,^16^ our stratified analyses were not adequately powered to assess potential effect modification. Future analyses should explore health-equity implications of right-to-counsel in greater depth.

## Conclusions

This cohort study found that phased rollout of tenant right-to-counsel in New York, New York, was associated with a significant reduction in adverse birth outcomes, including low birth weight and preterm birth. These findings suggest that Benefits of right-to-counsel might extend beyond the courtroom, promoting health at birth and across the life-course.

## Notes

Supplement 1. **eAppendix.** Difference-in-Difference Model Specification and Assumption Checking **eFigure 1.** Participant Recruitment Flowchart **eFigure 2.** Event Study Specification of Main Difference-in-Differences Model **eTable.** Primary Results and Sensitivity Analysis

Supplement 2.
Data Sharing Statement
