# Supplementary material for: Tenant Right-to-Counsel and Adverse Birth Outcomes in New York, New York
Source: JAMA Pediatr. 2024 Oct 28;178(12):1337–44. doi: 10.1001/jamapediatrics.2024.4699 (PMC11581741; doi:10.1001/jamapediatrics.2024.4699)
Supplement: Supplement 1. — eAppendix. Difference-in-Difference Model Specification and Assumption Checking eFigure 1. Participant Recruitment Flowchart eFigure 2. Event Study Specification of Main Difference-in-Differences Model eTable. Primary Results and Sensitivity Analysis [file jamapediatr-e244699-s001.pdf]

Supplemental Online Content

Leifheit KM, Chen KL, Anderson N, et al. Tenant right-to-counsel and adverse birth outcomes in New York, New York. *JAMA Pediatr*. Published online October 28, 2024.
doi:10.1001/jamapediatrics.2024.4699

eAppendix. Difference-in-Difference Model Specification and Assumption Checking
eFigure 1. Participant Recruitment Flowchart
eFigure 2. Event Study Specification of Main Difference-in-Differences Model
eTable. Primary Results and Sensitivity Analysis

This supplemental material has been provided by the authors to give readers additional information about their work.

© 2024 Leifheit KM et al. *JAMA Pediatrics.*

eFigure 1. CONSORT flow diagram

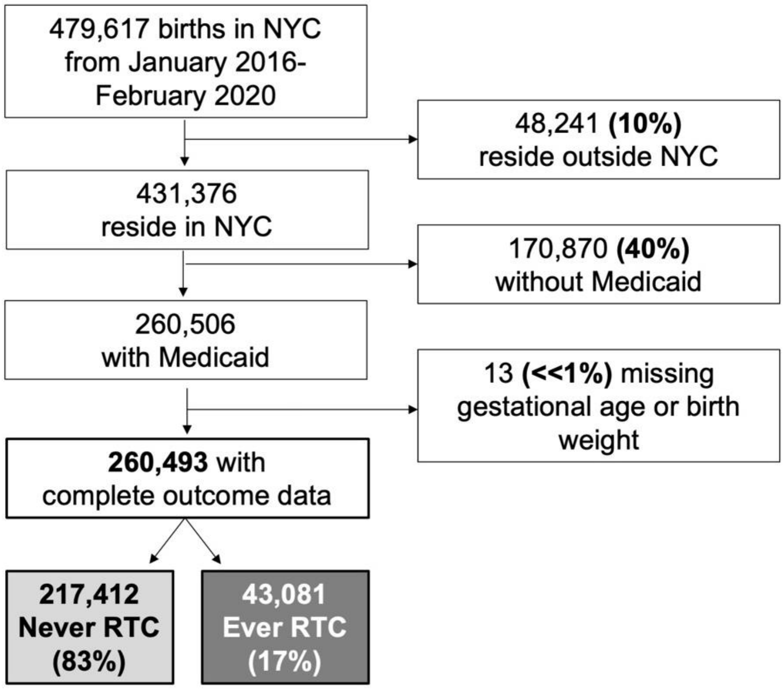

Appendix Figure 1. Specification of study sample

© 2024 Leifheit KM et al. *JAMA Pediatrics.*

I. Difference-in-Difference model specification and assumption checking

Our main regression analyses use a linear probability model with two-way fixed effects (i.e. dummy variables for ZIP code treatment units and time – calendar year and month). The unit of analysis is individual births, observed within ZIP codes and over time. Treatment is specified as a time-varying, lagged indicator of whether a ZIP code was treated with right-to-counsel 9 months prior to an infant's birth month. The indicator is coded zero for never-treated ZIP codes and pre-treatment ZIP codes; it is coded 1 post-treatment. A detailed model specification is included below:

$$\boldsymbol{Y_{ijk} = \beta_0 + \beta_1(RTC)_{j(k-9)} + \beta_2(ZIP)_j + \beta_3(Year)_k + \beta_4(Month)_k + \varepsilon_{jk}}$$

…where

Y = adverse birth outcome (dichotomous)
i = individual birth
j = ZIP code of residence at birth
k = year and month of birth
β_1 = difference-in-difference coefficient
β_2 = area fixed effects coefficient
β_3, β_4 = time fixed effects coefficients

In contrast to a canonical (two groups, two periods) difference-in-difference model, the two-way fixed effects approach allows for staggered treatment. This is necessary given that right-to-counsel was implemented in 3 distinct cohorts over the study period. As with a canonical difference-in-difference, two-way fixed effects control for 1) baseline differences between treated and untreated groups [via unit fixed effects, β_2 in our model] and 2) time trends that are common to both groups [via time fixed effects, β_3 and β_4 in our model].[1,2]

As with all difference-in-difference approaches, the foundational assumption of a two-way fixed effects model is that treated and untreated groups would have parallel or common trends in the absence of treatment. We assess this assumption be evaluating whether pre-treatment trends are parallel between the two groups. In our case, this means that pre-right-to-counsel trends in adverse birth outcomes in ZIP codes that go on to receive right-to-counsel should mirror trends in ZIP codes that are not treated. To check this assumption, we conduct an event study.[3] The event study is a more flexible version of the standard difference-in-difference model that allows treatment effects to vary across time, relative to treatment. To accomplish this, treatment is coded using a set of binary indicators representing leads and lags (i.e., months since a ZIP code was treated with right-to-counsel). For ZIP codes never treated during the study period, all binary indicators for leads and lags were set to zero. When event study coefficients are plotted (Figure 1), we can examine trends in pretreatment to assess potential violations of the parallel-trends assumption.[2] Visual inspection of the event study suggests that pre-treatment trends are parallel. We also conducted event studies for low birthweight and preterm birth, with very similar results.

Difference-in-difference analyses also implicitly assume static treatment effects. As a non-parametric approach, the event study also allows us to gauge whether treatment effects vary over time post-treatment. Relative to pre-treatment coefficients, post-treatment coefficients are lower, on average (suggesting that the difference in differences has narrowed), but there is no clear time trend in post-treatment coefficients. Thus, we conclude that the assumption of static treatment effects is reasonable and the difference-in-difference coefficient is a fair reflection of treatment effect dynamics.

---

[1] Wing C, Simon K, Bello-Gomez RA. Designing Difference in Difference Studies: Best Practices for Public Health Policy Research. Annu Rev Public Health. 2018 Apr 1;39:453-469. doi: 10.1146/annurev-publhealth-040617-013507. Epub 2018 Jan 12. PMID: 29328877.

[2] Wang, Guangyi; Hamad, Rita; White, Justin S. Advances in Difference-in-differences Methods for Policy Evaluation Research. Epidemiology 35(5):p 628-637, September 2024. | DOI: 10.1097/EDE.0000000000001755

[3] Goodman-Bacon A. Difference-in-Differences with Variation in Treatment Timing. National Bureau of Economic Research. Published online 2018. doi:10.1017/CBO9781107415324.004

© 2024 Leifheit KM et al. *JAMA Pediatrics.*

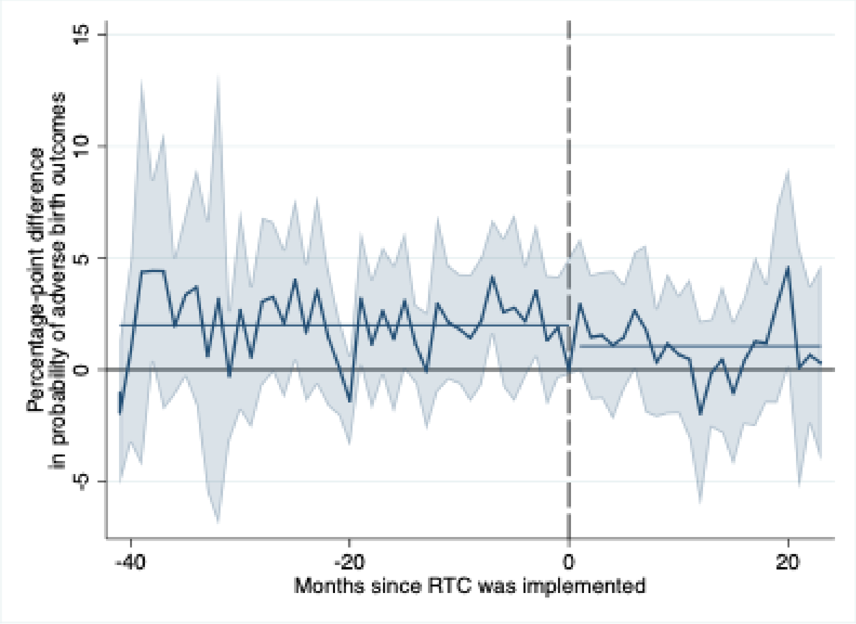

Figure 2. Event study specification of main difference-in-differences model. Horizontal lines indicate mean differences between right-to-counsel vs. control zip codes pre- and post-treatment.

A final assumption of the two-way fixed effects difference-in-difference model is that treatment effects are not heterogeneous by treatment group. In our case, this assumption would mean that right-to-counsel implementions in February 2017, October 2017, and October 2018 all had equal effects on adverse birth outcomes. To evaluate this assumption, we calculate unadjusted difference-in-difference estimates separately by phase (see Table 2) and also measure treatment effects separately by phase (see Appendix Table 2, *Alternate Exposure Definitions*). These analyses show qualitatively similar results across phases. Finally, we apply methods proposed by Callaway and Sant'Anna for a "heterogeneity-robust" difference-in-difference estimator. Because these methods require a standard panel dataset, we first collapsed individual birth outcomes to the ZIP code level (i.e. zip-month proportions of live births that were pre-term or low birthweight). The Callaway Sant'Anna approach produced an Average Treatment Effect on the Treated (ATT) point estimate that was identical to our main result (i.e. a 0.96 percentage-point reduction in adverse birth outcomes associated with right-to-counsel), although the result in this population-average model is no longer statistically significant. In exploring cohort-specific ATTs, there appears to be evidence of heterogeneity, with stronger reductions observed in the February 2017 and October 2018 cohorts and a null policy impact in the October 2017 cohort. Of note, at least one policy evaluation[4] found smaller relative increases in tenant representation in this implementation cohort, relative to the other two.

[4] Ellen IG, O'Regan K, House S, Brenner R. Do lawyers matter? Early evidence on eviction patterns after the rollout of universal access to counsel in New York City. Housing Policy Debate. 2021 Sep 3;31(3-5):540-61.

© 2024 Leifheit KM et al. *JAMA Pediatrics.*

III. Difference-in-Difference Model Sensitivity Analyses

Table. Primary results, N=260,439

Exposure	Outcome	Adjusted?*	Coefficient	95% CI	p-value
**Primary analyses**					
Right-to-counsel 9 months prior to birth	Adverse birth outcome (dichotomous)	N	-0.0096	-0.0184, -0.0009	0.031
	Low Birthweight (<2500 g, dichotomous)	N	-0.0073	-0.0141, -0.0006	0.032
	Preterm (<37 weeks, dichotomous)	N	-0.0091	-0.0171, -0.0010	0.028

Table. Results of sensitivity analyses, N=260,439 unless otherwise noted

Exposure	Outcome	Adjusted?*	Coefficient	95% CI	p-value
**Adjusted for birthing parent characteristics***					
Right-to-counsel 9 months prior to birth	Adverse birth outcome (dichotomous)	Y	-0.0098	-0.019, -0.0007	0.035
**Alternate exposure definitions**					
% of gestation exposed to right-to-counsel**	Adverse birth outcome (dichotomous)	N	-0.0072	-0.014, 0.0001	0.053
Trimester first exposed to right-to-counsel**		N			
Trimester 1			-0.0066	-0.0139, 0.0006	0.072
Trimester 2			0.0101	-0.0048, 0.0250	0.182
Trimester 3			0.0093	-0.0028, 0.0224	0.126
February 2017 phase only (later-treated ZIP codes excluded; N=236,655)	Adverse birth outcome (dichotomous)	N	-0.0092	-0.0181, -0.0003	0.042
October 2017 phase only (earlier and later-treated ZIP codes excluded; N=229,516)			-0.0095	-0.0289, 0.0098	0.332
October 2018 phase only (earlier-treated ZIP codes excluded; N=229,146)			-0.0122	-0.0337, 0.0093	0.265
**Alternate outcome definitions**					
Right-to-counsel 9 months prior to birth	Birthweight (g, continuous)	N	10.61	-4.11, 225.34	0.157
	Very Low Birthweight (<1500 g, dichotomous)	N	-0.0003	-0.0028, 0.0022	0.817
	Gestational age (weeks, continuous)	N	0.0350	-0.0206, 0.0906	0.216
	Very Preterm (<32 weeks, dichotomous)	N	-0.0009	-0.0035, 0.0016	0.473
**Stratified by birthing parent race and ethnicity**					
Hispanic (N=98,917)	Adverse birth outcome (dichotomous)	N	-0.0124	-0.0244, -0.0004	0.042
Non-Hispanic Asian / Pacific Islander (N=45,206)		N	-0.0052	-0.0212, 0.0109	0.526
Non-Hispanic white (N=55,264)		N	-0.0158	-0.0405, 0.0089	0.208
Non-Hispanic Black (N=76,552)		N	-0.0068	-0.0247, 0.0162	0.457

© 2024 Leifheit KM et al. *JAMA Pediatrics.*

**Collapsed and Heterogeneity-Robust Model Specifications**					
Collapsed by ZIP code (N=8,390)	Adverse birth outcome incidence (continuous)	N	-0.0128	-0.0245, -0.0010	0.033
Callaway and Sant'Anna DiD with multiple periods estimator (N=8,297)					
Overall ATT		N	-0.0096	-0.0400, 0.0208	0.537
February 2017 cohort			-0.0100	-0.0494, 0.0294	0.620
October 2017 cohort			0.0044	-0.0515, 0.0603	0.878
October 2018 cohort			-0.0417	-0.0898, 0.0064	0.090

*Adjusted for individual-level covariates: birthing parent age, race/ethnicity, education, nativity, marital status, and parity
** In contrast to our main primary specification, which requires 9 or more months of treatment, these specifications allow for partial treatment (i.e. RTC implemented during pregnancy)

© 2024 Leifheit KM et al. *JAMA Pediatrics.*
