# Supplementary material for: Tenant Right-to-Counsel and Adverse Birth Outcomes in New York, New York
Source: JAMA Pediatr. 2024 Oct 28;178(12):1337–44. doi: 10.1001/jamapediatrics.2024.4699 (PMC11581741; doi:10.1001/jamapediatrics.2024.4699)
Supplement: Supplement 2. — Data Sharing Statement [file jamapediatr-e244699-s002.pdf]

Data Sharing Statement

Leifheit. Tenant Right-to-Counsel and Adverse Birth Outcomes in New York, New York. *JAMA Pediatr*. Published October 28, 2024. doi:10.1001/jamapediatrics.2024.4699

Data
Data available: No

Additional Information
Explanation for why data not available: Data belong to the New York City Dept of Health and Human Hygiene. We are not permitted to share per our data use agreement. We can share analytic code and advise on how to apply for data.
